# Potential Short- and Long-Term Physiological Effects of Ischemic Preconditioning as an Ergogenic Aid: Revisiting Foundational Mechanisms and Applications

**DOI:** 10.1007/s40279-025-02232-3

**Published:** 2025-05-21

**Authors:** Moacir Marocolo, Hiago L. R. Souza, Pia Surke, Alexander Ferrauti

**Affiliations:** 1 Department of Training and Exercise Science, Faculty of Sport Science, Ruhr University Bochum, Bochum, Germany; 2 Integrated Laboratory of Physiology and Performance (LABIFID), Department of Biophysics and Physiology, Federal University of Juiz de Fora, Juiz de Fora, Minas Gerais Brazil

## Abstract

Ischemic preconditioning (IPC) has emerged as a promising intervention for enhancing health- and exercise-related outcomes. Initially recognized in the 1980s and 1990s for its cardioprotective effects in clinical and animal studies, IPC has since garnered attention for its potential ergogenic benefits. Despite growing interest, the underlying physiological mechanisms remain poorly understood, leading to research exploring cause–effect relationship and evaluating IPC efficacy across diverse exercise models, often yielding mixed results. This Leading Article aims to clarify proposed mechanisms by which IPC may enhance athletic performance and facilitate healing effects. Specifically, this Leading Article discusses both the immediate (short-term) and sustained (long-term) effects of IPC. Short-term effects primarily involve acute improvements in vascular function and exercise capacity, while long-term effects may include cumulative benefits such as enhanced recovery, mitigation of exercise-induced muscle damage and adaptative physiological responses. This article highlights the importance of optimizing experimental protocols by extending the time window between IPC application and testing, to maximize performance outcomes, particularly under conditions associated with muscle damage. Future research should prioritize exploring the long-term effects of IPC on performance and recovery to better understand its potential as a reliable ergogenic aid.

## Key Points

**Table:** 

Ischemic preconditioning (IPC) as an ergogenic aid remains poorly understood in terms of its underlying physiological mechanisms. Enhancing our knowledge from this perspective is crucial for clarifying IPC’s role as a performance-enhancing intervention.
Potential mechanisms by which IPC could improve athletic performance or healing processes are believed to have both short- and long-term effects, including enhancements of blood cell deformability, vasodilation, blood flow, mitochondrial activity, and reductions in oxidative stress and inflammatory response.
Although extensively demonstrated in clinical settings, the protective effects of IPC and its applications in sports science remain overlooked. The potential protective effects of IPC on exercise-induced muscle damage warrant further investigations.

## Introduction

Brief periods of occlusion and reperfusion of blood flow, known as ischemic preconditioning (IPC), have been shown to improve both health- and exercise-related outcomes [1–3]. Initially recognized for its protective effects in cardiac tissue, IPC gained prominence in the 1980s and 1990s through clinical and experimental studies [4–6], which later demonstrated its benefits in the liver [7], kidneys [8], brain [9], intestine [10], and skeletal muscle [11]. These findings underscore IPC's potential as a versatile tool for improving physiological resilience and performance. However, as early as the 1950s, non-invasive studies using intermittent cuff administration for occlusion and reperfusion of blood flow aimed at enhancing muscle performance through hyperemia [12–14]. Nukada [14] reported improved calf muscle performance after blood flow occlusion, corroborated by Muller [13] in 1958, but not by Collier and Percival [12] soon after. While these studies proposed that reactive hyperemia would augment blood, oxygen, and energy delivery to peripheral muscles, Muller concluded that it primarily enhances the outflow of metabolic waste products from muscle metabolism into the bloodstream [13].

In the context of health, IPC (sufficient cuff-pressure to induce blood flow occlusion) has consistently demonstrated efficacy in reducing damage and improving protection to various tissue types, such as brain [15–17], endothelium [18, 19], kidney [20], and myocardial and skeletal muscle [6, 21, 22], with evidence suggesting also a sex-dependent effect [23, 24], being more favorable in females compared to males. The heart exhibits increased resistance to prolonged ischemia following IPC [4, 25], attributed to reduced mitochondrial apoptosis and lower production of reactive oxidative species (ROS) [26, 27]. Additionally, animal experiments have shown that IPC demonstrated preservation of efferent sympathetic and vagal autonomic nerve responses [28], and protection against skeletal muscle infarction [29]. Clinical studies in humans corroborate these findings, demonstrating reduced pain following total knee arthroplasty [26], attenuation of reperfusion injury in patients undergoing arm IPC prior to percutaneous coronary intervention [30], and decreased serum T-troponin levels up to 48 h after elective coronary artery bypass surgery [31].

In the field of sports science, the effects of IPC on exercise performance have been systematically explored [32, 33]. Since its initial proposal as a potential ergogenic aid in the early 2000s [34], interest in IPC has grown significantly, with an increasing number of publications investigating its application. While clinical evidence underscores IPC’s protective effects on muscle tissue and its ability to enhance blood flow and oxygen delivery, the theoretical basis for its use as an ergogenic aid remains insufficiently clarified. Most studies adopt a cause–effect model, examining the efficacy of acute IPC interventions on various exercise protocols or sport modalities. However, these investigations often lack robust exploration of the underlying physiological mechanisms, leading to inconsistent findings.

Proposed mechanisms for IPC´s ergogenic effects frequently rely on indirect evidence [33], such as changes in blood flow, biomarkers, or other physiological responses, without directly linking these changes to observed performance or recovery outcomes [33]. Direct evidence would necessitate controlled mechanistic studies that isolate specific pathways, such as IPC-induced signaling cascades (e.g., hypoxia-inducible factor 1-alpha, HIF-1α and peroxisome proliferator-activated receptor gamma coactivator 1- alpha, PGC-1α) [35] or the direct demonstration of causal relationships between the intervention and its purported effects. This gap in mechanistic understanding may partly explain the divergent findings and ongoing debates surrounding IPC's efficacy.

Recent evidence suggests that IPC elicits both short (acute) and longer-term effects [36, 37]. Acute responses, occurring within 10 min to 4–6 h post-intervention [36], are associated with improved endothelial function, transient vasodilation, and reduced oxidative stress, which collectively enhance blood flow, mitigate inflammation, and minimize muscle fiber damage [38, 39]. In contrast, long-term effects, defined as occurring within 12–24 h and extending up to several days [36, 37], include the initiation of angiogenesis, prevention of myofibrillar disorganization, and increased mitochondrial activity [40]. These adaptations, traditionally considered to require successive IPC applications, may emerge earlier than anticipated, contributing to recovery and performance enhancements within the 24–48-h window.

Thus, to address these knowledge gaps, we propose a rational mechanistic framework for IPC's effects on exercise performance and recovery. This framework emphasizes the time-dependent nature of IPC-induced adaptations and highlights their potential to bridge the gap between acute recovery benefits and the initiation of structural changes that support long-term performance outcomes. Additionally, we have suggested further directions for research on this topic.

## Current Supporting Evidence for IPC as an Athletic Ergogenic Aid

The premise that IPC enhances blood flow and oxygen delivery during the reperfusion phase after cuff release [41, 42] would suggest that a more plausible effect would be observed in activities more dependent on the cardiovascular system rather than those primarily related to strength. However, increased blood flow, compared to the baseline level, during the hyperemia phase after cuff release generally lasts about 60 s [42], normally followed by the same pattern of local muscle oxygenation [41, 43]. Nevertheless, IPC has been shown to enhance performance and recovery across various modalities. Specifically, it improves cycling performance by increasing the maximal workload achieved [44] and power output [45], increases muscle oxygenation during sustained isometric exercise [46], and reduces the rate of torque loss [47] as well as prolonging time to exhaustion [48] and accelerating recovery [32, 49]. Additionally, IPC has been demonstrated to enhance performance in activities, such as improving completion times in 1-km and 3-min kayak time trials [50], as well as in longer-duration exercises, such as performance in 5-km time trials [51]. Although studies have shown divergent results, ranging from beneficial [44], null [52], to detrimental [53] effects, the potential ergogenic effects of IPC are supported by evidence of both short-term responses, such as immediate improvements in blood flow and muscle oxygenation, and long-term adaptations, such as enhanced recovery and performance over repeated sessions. These effects suggest IPC’s relevance across different time scales of athletic preparation.

### Short-Term IPC Performance Enhancement Effects: Neural Afferent-Efferent Drive and Vascular Changes

The hyperemia phase following last cuff release after IPC induces a transient increase in blood flow, lasting approximately 60 s [42], before returning to baseline values within 100 s [54]. Similarly, local muscle oxygenation briefly rises after IPC, peaking immediately post-reperfusion and reverting to baseline values within a similar timeframe [55]. These short-term physiological responses suggest that the interval between IPC application and the onset of exercise or testing, as well as its duration, should be kept minimal (within the aforementioned timeframes) to optimize potential benefits from blood flow, muscle oxygenation, and performance [3, 56]. However, in practical settings, particularly during moderate- to severe-intensity activities (i.e., activities performed below or above the second ventilatory threshold/lactate threshold), a warm-up is typically required. This additional preparatory phase would prolong this interval between IPC and exercise, potentially negating the transient vascular and oxygenation enhancements, as these values return to baseline prior to activity onset. Interestingly, a recent review reported that among IPC papers investigating performance, 54 utilized an interval of up to 30 min between IPC protocol and the test, 14 employed intervals ranging from 31 to 60 min, and only four extended beyond 60 min between the final IPC cuff cycle and the exercise or test [57].

Numerous vascular responses to IPC have been described, including contralateral artery vasodilation observed at rest [58] and during 30 min of rhythmic handgrip exercise [59], the preservation of brachial artery flow-mediated dilation following a heavy-intensity running protocol (5-km time trial) [38], increased vascular conductance [60], elevated levels of eNOS-derived nitrite, and improved regulation of mitochondrial respiration within endothelial cells [61]. Furthermore, increased cutaneous vascular conductance and artery flow-mediated dilation have been observed up to 9 days after a 1-week repeated IPC intervention [62] and, although not measured during exercise performance, may enhance the delivery of oxygen and nutrients to working muscles, particularly during heavy- and severe-intensity exercise. Additionally, they may improve the removal of metabolic by-products such as lactate and hydrogen ions [51], which are particularly relevant when exercise is performed at or above the critical power threshold.

IPC also has been shown to influence muscle oxy- and deoxygenation [46, 63] and muscle oxidative recovery kinetics [64]. While much of the available NIRS-derived evidence suggests greater muscle deoxygenation during moderate- to severe-intensity exercise [63], this reflects increased oxygen utilization by active muscles [65]. Contrary to interpretations suggesting reduced desaturation, evidence indicates enhanced oxygen extraction capacity [65], likely mediated by improved vascular function and increased release of vasoactive compounds, such as nitric oxide [60]. Additionally, IPC has been associated with prolonged apnea time [63], which may reflect improved tolerance to hypoxia and enhanced oxygen utilization efficiency [66, 67]. This combined evidence highlights the complex interplay between oxygen delivery and extraction mechanisms following IPC, particularly as exercise intensity escalates from light to severe domains.

It has also been demonstrated that IPC increases red blood cell (RBC) deformability, which may significantly improve microcirculation. This increase in RBC deformability occurs within a short timeframe of approximately 15 to 40 minutes after IPC [68] at rest or when individuals undergo an incremental test leading into severe-intensity domains [69]. RBC deformability is crucial for athletic performance [70, 71] as it may reduce cells’ diameter, facilitating their passage through narrow capillaries and enhancing overall microcirculation [69, 72]. Furthermore, combining IPC with regular exercise (from moderate- to severe-intensity domains) training amplifies the benefits on RBC deformability [73]. These combined effects of vasodilation and improved microcirculation may also significantly improve the long-term beneficial adaptations, including the activation of antioxidative and anti-inflammatory pathways. Enhanced microcirculation ensures efficient delivery of oxygen and nutrients while facilitating the removal of metabolic waste, which is crucial for reducing oxidative stress and inflammation, particularly in higher intensity exercise scenarios.

The aforementioned IPC-induced changes may be mediated by central and vascular autonomic activity [74, 75] as well as linked to neural and humoral factors [1, 76]. In terms of humoral pathways, several humoral factors appear to play a role in the protective effects of IPC. Substances such as adenosine, bradykinin, prostaglandins, opioids, endocannabinoid, and others have been identified [77–80], as well as calcitonin gene-related peptide, which appear to function via activation of PKC and may be released by IPC [1, 81]. Regarding neural signaling, although some mechanisms remain unclear, it has been proposed that IPC influences neuromuscular fatigue resistance in heavy- to severe-intensity exercise [82]. In addition, emphasis has been placed on the necessity of a neural/central-peripheral integration pathway underlying IPC molecular or physiological responses [83], and supported the proposition of IPC responders and non-responders [37]. The concept of responders versus non-responders highlights the importance of investigating individual variability in IPC efficacy, which may arise from complex interactions among physiological, molecular, and neural factors.

**Fig. 1:**
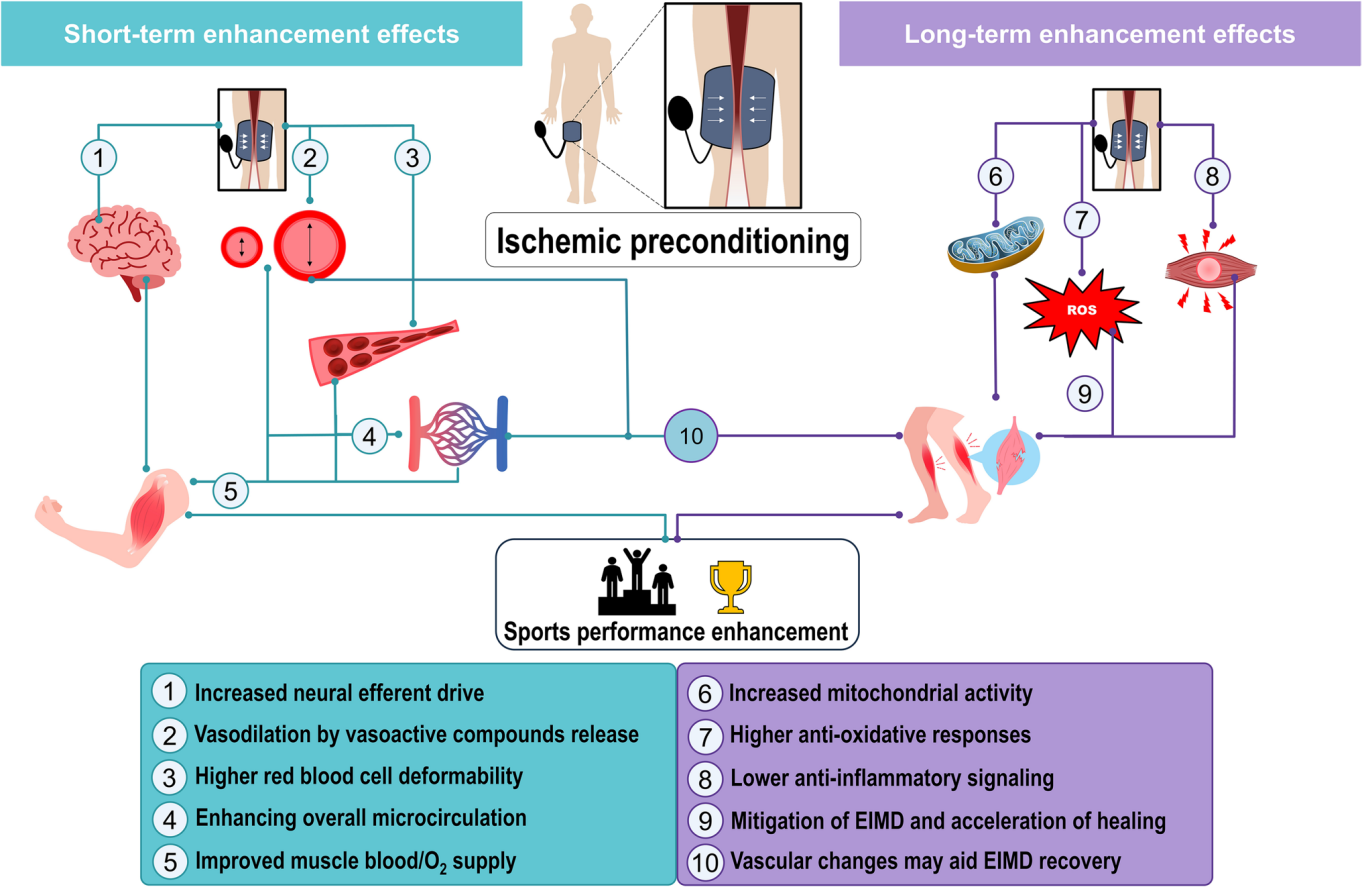
Proposed mechanisms by which IPC could improve athletic performance and recovery. The left side of the figure depicts short-term effects encompassing alterations in bottom-up afferent and increases in top-down efferent drive by cuff-pressure, enhancements of red blood cells deformability as well as vasodilation-inducing vasoactive substances, optimizing blood flow in the microcirculation and facilitating a greater supply of oxygen and nutrients, as well as the removal of CO_2_ and metabolic waste. The right side of the figure outlines the long-term effects, which are associated with a signaling cascade that increases mitochondrial activity while reducing oxidative stress and the inflammatory response. The sequential occurrence of these factors could mitigate EIMD and accelerate the healing process, thereby optimizing athletic performance. Notably, short-term vascular effects (10), although transient, could enhance EIMD healing in a post-exercise condition. *EIMD* exercise-induced muscle damage, *IPC* ischemic preconditioning, *ROS* reactive oxidative species

In this context, when the low-pressure cuff intervention group (placebo or sham condition) is included in IPC experimental designs, non-significant or very low-magnitude differences between IPC and placebo-sham are observed [33, 84]. This observation suggests that a portion of the reported IPC effects in many studies may not solely result from physiological modifications but could also be influenced by placebo-related psychophysiological responses. Placebo effects are mediated through complex neurobiological processes, including the release of endogenous opioids and dopamine, which involve central-peripheral (bottom-up and top-down) afferent and efferent integration facilitated by the nervous system, triggered by the cuff pressure [83]. In this context, the cuff pressure may trigger afferent feedback from group III/IV muscle afferents, potentially leading to a reduction in inhibitory supraspinal control, enhancing efferent neural drive and motor cortex activation. This proposal aligns with Fig. 1 and adds a novel mechanistic perspective to IPC´s short-term effect. It is important to emphasize that only the IPC intervention (high-pressure cuff) is likely to produce genuine physiological or vascular effects, whereas the sham or placebo intervention (low-pressure cuff) is considered inert from a physiological standpoint. However, it may still be capable of promoting improvements depending on expectations due to the prior verbal instructions given [85].

While evidence supports the effectiveness of IPC administration, repeated IPC sessions over consecutive days do not appear to provide additional benefits compared to a single acute session, particularly for short-term recovery following severe-intensity exercise [86]. Furthermore, the potential adaptation-induced effects of IPC-cuff administration, whether using high or low pressure, may diminish with repeated use. This phenomenon is exemplified by the progressive decline in the positive effects of IPC on high-intensity resistance-arm exercise performance over time [87]. The left side of Fig. 1 schematically illustrates the proposed short-term neural and vascular effects of IPC on sports performance. Although the vascular effects of IPC are typically short term, they can contribute to more pronounced long-term responses, including enhanced mitochondrial activity, increased antioxidant and anti-inflammatory effects, reduced muscle damage, and improved healing. These benefits may arise either directly through preconditioning effects or indirectly by influencing post-exercise recovery mechanisms, particularly in heavy- and severe-intensity exercise domains.

### Long-Term IPC Effects: Anti-oxidative and Anti-inflammatory Responses Mediating Muscle Healing Pathways

In addition to the short-term effects, the use of IPC can alter indirect pathways contributing to improved performance. Acute as well as repeated IPC application may diminish redox signaling following subsequent exercise, resulting in decreased inflammation pathway cascades [88], and acutely upregulating endogenous antioxidant defenses in muscle tissue within minutes after blood flow interruption [89, 90]. This process involves the increased expression of antioxidant enzymes, including superoxide dismutase, glutathione peroxidase, and catalase, which work to scavenge ROS generated during exercise. By reducing oxidative stress, these enzymes aid in mitigating cellular damage and preserving muscle function, thereby supporting recovery and performance [91]. In addition, the release of NO and other vasoactive compounds (e.g., endothelin-1, prostacyclin, adenosine) within the 3–4 h after an IPC event [92–95] reduces vascular resistance and may increase circulation and microcirculation [93–96]. Also, adenosine has been shown to enhance the microvascular hyperemic response [97], along with bradykinin [94, 95, 98], which is also known for its mediated inflammatory action and cardioprotective effects following ischemia [99]. These effects could play a role in the long-term effects.

IPC elicits an anti-inflammatory response to exercise by reducing the expression of pro-inflammatory cytokines such as interleukin-6 (IL-6) and tumor necrosis factor alpha (TNFα), and may contribute to reduced muscle damage and expedited recovery [100]. Hence, IPC may activate a variety of intracellular signaling pathways associated with cell survival and adaptation to stress. These pathways encompass the phosphatidylinositol-3-kinase (PI3K)/Akt pathway and the mitogen-activated protein kinase (MAPK) pathway [101], which govern processes including cell proliferation, protein synthesis, and apoptosis. Such prior activation may foster cellular resilience and minimize muscle damage in a high-intensity effort and/or aid healing for a subsequent exercise or competition [102]. The right side of Fig. 1 shows long-term IPC effects related to anti-oxidative and anti-inflammatory pathways on reducing muscle damage, improving healing processes, and consequently enhancing sports performance.

## Effects of IPC on Exercise-Induced Muscle Damage Healing: Revisiting Foundational Mechanisms and Applications

While the protective effect of IPC against sustained ischemia events has been extensively demonstrated in various tissues such as heart [103], brain [104], and liver [105], its potential application in attenuating exercise-induced muscle damage (EIMD) remains overlooked in sports science, warranting properly designed studies. However, prolonged ischemia, such as during orthopedic surgeries with tourniquet use, can cause muscle damage, including lysosome accumulation, edema, and fiber necrosis, even within 90 min. Conversely, shorter IPC protocols (e.g., 1–4 cycles of 3–5 min of ischemia) can promote beneficial effects, such as reducing EIMD and enhancing recovery. This highlights the need for careful protocol standardization in research and practice.

After nearly 40 years of evidence demonstrating a significant reduction in myocardial injury by approximately 75% [4], only a limited number of studies have explored the potential application of IPC for protective effects in the field of sports science [106–108]. Previous research did not find differences in muscle creatine kinase (CK) (a biomarker indicator of muscle damage) or performance when comparing IPC and placebo, even across short protocols such as ten sets of 12 repetitions [107] or longer three sets of 100 repetitions [108] in a knee eccentric isokinetic protocol. Conversely, acute (3 × 5 min at 220 mmHg) and repeated IPC (3 days × 3 × 5 min at 220 mmHg) application improved recovery time (24–72 h) of maximal voluntary isometric contraction without significant changes in CK levels after an EIMD drop-jump protocol [106]. Nevertheless, when applied prior to an eccentric exercise biceps curl, IPC effectively reduced CK levels at 24 and 48 h post-exercise compared to no cuff intervention [109]. On the other hand, post-exercise ischemic conditioning (i.e., an IPC intervention administered after exercise) did not affect CK responses, despite the observed improvement in performance recovery in a cycling time-to-exhaustion test [110].

In addition to these studies, a proposed mechanism for IPC-induced muscle healing may involve both morphological adaptations and intracellular signaling changes. Morphologically, IPC may promote angiogenesis, which enhances blood vessel formation, thereby improving circulation in damaged skeletal muscle [111]. This increased circulation facilitates the delivery of oxygen and nutrients while aiding in the removal of metabolic waste products, promoting an optimal environment for tissue repair. Furthermore, IPC may enhance myofibrillar organization, leading to improved alignment and structural integrity of muscle fibers [111]. Together, these adaptations may support recovery, reduced muscle damage, and more effective healing. Additionally, IPC promotes enhanced mitochondrial activity, linked to increased energy production, reduced ROS generation, higher partial oxygen pressure (and availability to the muscles) and greater enzymatic density, by improving the content of enzymes like citrate synthase and cytochrome C oxidase [96]. In addition to the abovementioned adaptations, IPC may enhance muscle healing by influencing cellular and molecular pathways. One proposed mechanism involves the enhanced activity of membrane ion channels, such as potassium and calcium channels [1, 2, 112, 113], which are critical for maintaining cellular homeostasis under stress conditions. IPC-induced modulation of these ion channels may help stabilize cell membrane potential, reduce calcium overload, and prevent cellular damage, creating a more favorable environment for muscle repair. Furthermore, IPC has been shown to suppress proinflammatory signaling cascades including the expression of proinflammatory gene expression in circulating leukocytes, and reduce apoptosis in damaged tissues [114]. These effects collectively dampen the inflammatory response, limiting secondary tissue damage and promoting an anti-inflammatory milieu, conducive to healing. By minimizing excessive inflammation and apoptosis, IPC may facilitate the resolution of inflammation and accelerate tissue regeneration, improving muscle recovery following injury or surgery [114, 115]. It is important to highlight that vasodilation and greater vascular conductance [62], despite being triggered acutely, can persist for a longer time window [62] and significantly optimize the pathways and signaling of the long-term effects, such as the enhancement of anti-inflammatory and anti-oxidative effects and anti-oxidative effects, thereby contributing to the healing of muscle damage.

## What Remains to be Investigated in IPC Experiments in Sports Science?

Examining a straightforward cause-and-effect relationship, where a predetermined IPC protocol with three or four cycles of occlusion and reperfusion is applied before a warm-up preceding a performance test, addresses only a fraction of the experimental conditions that should be considered. Factors such as cuff protocols, sporting modalities, the fitness level of participants, the timing between cuff application and exercise, and verbal instructions should also be carefully considered, as they can all influence the outcomes and mechanisms of IPC [116]. This variability in conditions across studies may contribute to differences in response rates, and it is important to strike a balance between variability and homogeneity when designing IPC-based experimental studies. Additionally, sex must be considered as a critical variable, as physiological differences between males and females could influence the response to IPC and its subsequent effects on performance [117, 118]. However, studies involving females remain limited [57].

Given the vast domain of variables and experimental conditions within this field, providing guidance for future investigations is highly valuable. Establishing a precise IPC protocol, such as three or four cycles of 5 min each, and focusing on its primary impact on muscle protection is a judicious course of action. Prioritizing exercise protocols that consistently induce muscle damage offers a relevant context of assessing IPC’s dual role in enhancing recovery and mitigating muscle damage, potentially broadening its applications beyond acute performance enhancement. Alongside measuring damage markers, it is crucial to assess physiological variables (e.g., power output, force, velocity) during performance tests and recovery phases, together with subjective scales (rate of perceived exertion and recovery), ensuring a comprehensive evaluation of the effects. Since the acute administration of IPC may produce effects of small magnitude that are often difficult to detect, investigating its impact under conditions of muscle damage could enhance its efficacy by leveraging mechanisms that are more directly involved in tissue repair and recovery. Additionally, it is important to always measure occlusion pressure when applying the IPC maneuver. This ensures that the cuff pressure parameters, and the corresponding perception of the individual being tested, remain consistent, preventing potential alterations in the performance response.

## Conclusion

Considering the diverse time- and intensity-response characteristics of physiological systems and the varied molecular and cellular signaling pathways triggered by IPC, its effects may not immediately manifest, but rahter become evident within a 12- to 24-h window, corresponding to long-term adaptations [86, 119]. This temporal aspect must be carefully accounted for in experimental designs aimed at identifying optimal time points for IPC-induced responses. Consequently, performance measurements should be compared at later intervals or across multiple time points to capture the full spectrum of IPC’s effects. Notably, delayed IPC-induced changes have been well-documented [120, 121]. Given that many sports science studies often employ short intervals—typically less than 60 min—between IPC application and subsequent exercise or testing [57], there is a critical need to reevaluate this prevalent research approach.
